# A label-retaining but unipotent cell population resides in biliary compartment of mammalian liver

**DOI:** 10.1038/srep40322

**Published:** 2017-01-13

**Authors:** Janeli Viil, Mariliis Klaas, Kadri Valter, Denis Belitškin, Sten Ilmjärv, Viljar Jaks

**Affiliations:** 1 Institute of Molecular and Cell Biology, University of Tartu, Tartu, Estonia; 2 Department of Pathology and Immunology, Medical School, University of Geneva, Geneva, Switzerland; 3 Karolinska Institutet, Stockholm, Sweden

## Abstract

Cells with slow proliferation kinetics that retain the nuclear label over long time periods–the label-retaining cells (LRCs)–represent multipotent stem cells in a number of adult tissues. Since the identity of liver LRCs (LLRCs) had remained elusive we utilized a genetic approach to reveal LLRCs in normal non-injured livers and characterized their regenerative properties *in vivo* and in culture. We found that LLRCs were located in biliary vessels and participated in the regeneration of biliary but not hepatocyte injury. In culture experiments the sorted LLRCs displayed an enhanced self-renewal capacity but a unipotent biliary differentiation potential. Transcriptome analysis revealed a unique set of tumorigenesis- and nervous system-related genes upregulated in LLRCs when compared to non-LRC cholangiocytes. We conclude that the LLRCs established during the normal morphogenesis of the liver do not represent a multipotent primitive somatic stem cell population but act as unipotent biliary progenitor cells.

The regenerative potential in adult tissues is commonly attributed to a rare population of tissue-specific somatic stem cells. Mammalian liver possesses an extraordinary regenerative capacity^1^ and there has been a long-standing view that potential liver stem/progenitor cells are located in the smallest biliary vessels - the canals of Hering (reviewed in ref. 2). The biliary origin of liver progenitors was further supported by findings that liver regeneration is often accompanied by the appearance of proliferative biliary cells with characteristic oval nuclei–the oval cells. In addition, cells bearing biliary markers have been shown to possess enhanced regenerative properties (reviewed in ref. 3). However, this concept has been recently challenged by a series of *in vivo* lineage tracing experiments, which demonstrate that a subset of hepatocytes might be the source of bipotent progenitor cells that contribute to the two liver parenchymal compartments - the hepatocytes and biliary cells^4^,^5^,^6^,^7^,^8^,^9^,^10^.

Somatic stem cells are characterized by their ability to self-renew, the ability to regenerate all cell types in a given tissue and relative proliferative quiescence. Consequently, the retention of nuclear DNA label has been used as a measurement of slow proliferation rate to identify potential tissue-specific stem cells termed the label-retaining cells (LRCs)^11^. Prior to the era of advanced mouse genetics a pulse administration of nucleotide analogues such as tritiated thymidine (^3^H-thymidine) or 5-bromo-2′-deoxyuridine (BrdU) followed by a chase period was used to identify quiescent cells in various tissues. Such approach was used to identify potential stem cells in oral mucosa and epidermis^11^, the intestine^12^, corneal limbus^13^, hair follicles^14^, mammary gland^15^ and hematopoietic system^16^. However, the need for tissue processing for detecting the nuclear label excluded the possibility to directly isolate live LRCs and their closer characterization. Only the use of genetically modified mice facilitated the isolation and extensive characterization of LRCs from hair follicles, hematopoietic tissue, kidney, mammary gland, intestine, thymic epithelium, prostate and submandibular gland^17^,^18^,^19^,^20^,^21^,^22^,^23^,^24^.

Although there is evidence that liver contains LRCs^25^,^26^ their cellular identity and contribution to liver repair has been an open question. We hypothesized that liver LRCs (LLRCs) might act as primitive liver progenitor cells and aimed to study their role in liver maintenance and regeneration. Since the formation of biliary tracts–the potential liver stem cell niches–occurs only peri- and postnatally^27^, we induced the expression of histone 2B–enhanced green fluorescent protein (H2B-EGFP) fusion protein in the liver cells of newborn pups and chased the label until the maturation of liver. The LLRCs were clustered in portal areas in biliary ducts and expressed biliary and oval cell markers. Moreover, the LLRCs were induced to proliferate upon biliary but not upon hepatocyte injury and formed colonies of cells bearing only biliary but not hepatocyte markers in culture. Furthermore, lineage tracing of K19-expressing biliary cells revealed no contribution from biliary compartment to hepatocytes in any of the six different liver injury models tested, demonstrating that liver biliary cells do not participate in hepatocyte regeneration. Taken together, we demonstrated for the first time that the LLRCs established during normal liver morphogenesis act as unipotent biliary progenitor-like cells.

## Results

### The liver label-retaining cells reside in bile ducts and express biliary and liver progenitor cell markers

**Figure 1:**
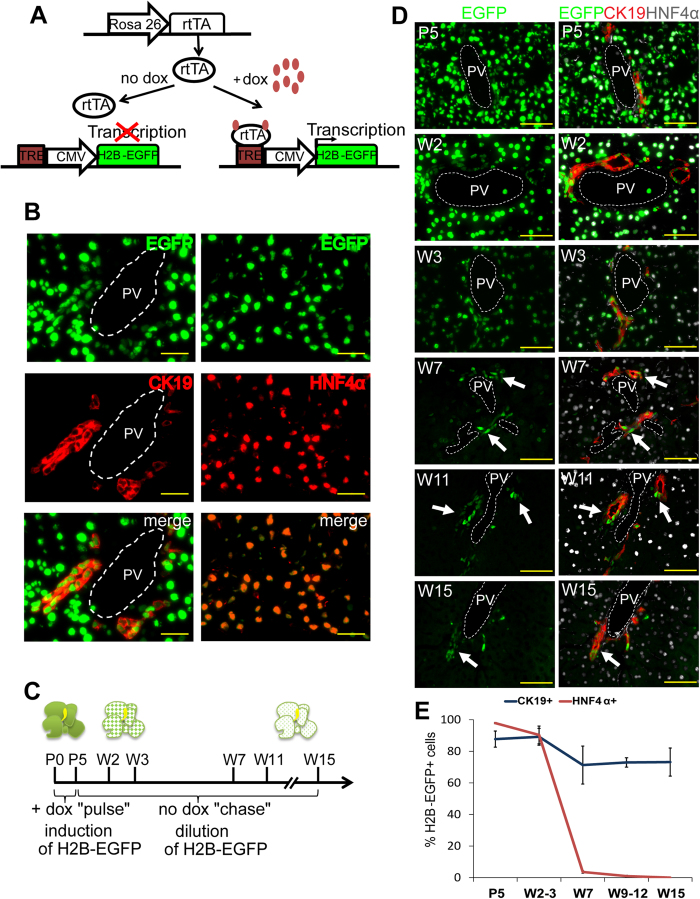
Liver LRCs are located in bile ducts. (**A**) Principle of genetic LRC labeling approach. In the absence of dox rtTA does not bind to TRE and H2B-EGFP is not expressed. Dox treatment results in transcriptional activation of H2B-EGFP. (**B**) H2B-EGFP (green) expression in biliary compartment (left) and hepatocytes (right) in P5 liver after 5 days of dox treatment. Scale bars: 25 μm. (**C**) Schematic representation of pulse-chase experiment. Bigenic pups received dox from P0–P5. H2B-EGFP expression was monitored at indicated time points. (**D**) Establishment of LRCs. Arrows point to CK19 positive (red) LRCs. Scale bars: 50 μm. (**E**) Loss of EGFP signal in hepatocytes (HNF4α+, red) and cholangiocytes (CK19+, blue) during 15 weeks. n = 3 per time point. Data represent mean ± SEM. PV-portal vein.

To identify LRCs in adult liver we took advantage of a bitransgenic mouse model where the expression of H2B-EGFP fusion protein was controlled by the presence of tetracycline analog–doxycycline (dox). To generate such system we bred mice harboring a reverse tetracycline-dependent transactivator expression cassette inserted into ubiquitously active Rosa26 locus (R26-rtTA)^28^ and a mouse line containing the H2B-EGFP expression construct controlled by the tetracycline response element (TRE) (Fig. 1A)^19^. As anticipated, addition of dox to the drinking water of nursing females induced the expression of H2B-EGFP in biliary cells and hepatocytes of bigenic pups (Fig. 1B).

Since biliary vessels that start to differentiate at E17,5, are formed as independent organs only perinatally and the maturation of hepatocytes takes place only after birth^1^,^27^ we assumed that the potential label-retaining tissue-specific stem cell compartments are formed during the postnatal period. As the proliferative activity in mouse livers declined constantly after birth (Supplementary Fig. S1A–D) we administered dox during P0–P5 to achieve maximum labeling of the liver cells and monitored the decrease in nuclear H2B-EGFP labeling for 15 weeks (Fig. 1C). While the intensity of the EGFP signal gradually declined in the majority of liver cells, patches of cells that retained H2B-EGFP label became apparent in portal areas after the age of 7 weeks (Fig. 1D and Supplementary Fig. S2A).

**Figure 2:**
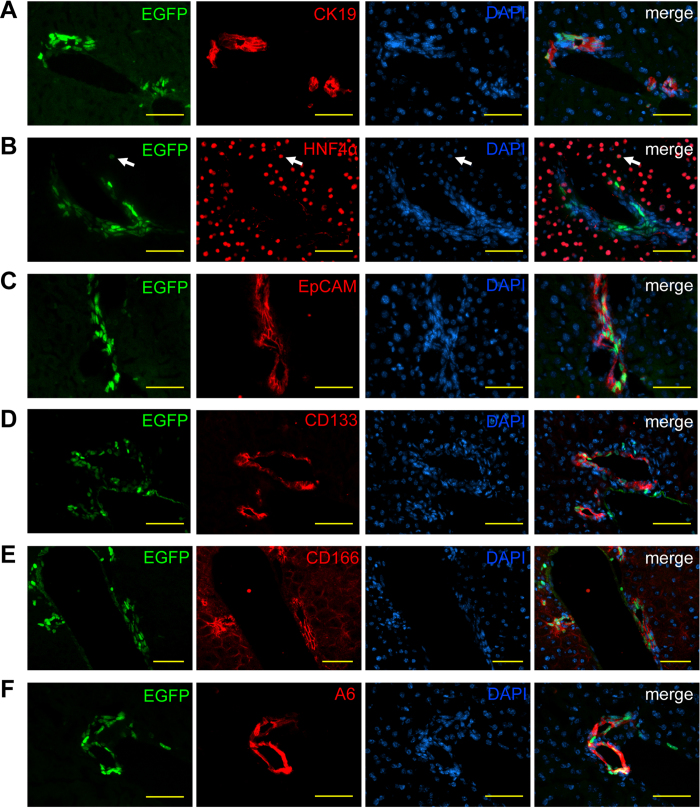
LLRCs express biliary and progenitor cell markers. (**A**–**F**) Frozen liver sections from 9–11 weeks old mice were immunostained with (**A**) CK19, (**B**) HNF4α, (**C**) EpCAM, (**D**) CD133, (**E**) CD166, and (**F**) A6 antibodies (red) and DAPI (blue). LRCs (green) in the bile ducts were positive for CK19, EpCAM, CD133, CD166 and A6. Rare LRCs in the parenchyma were HNF4α positive (**B**, arrow). Scale bars: 50 μm. n = 3.

Staining the sections with antibodies recognizing biliary epithelial marker CK19 (cytokeratin 19) and hepatocyte marker HNF4α (Hepatocyte Nuclear Factor 4, Alpha) revealed that the majority of LLRCs localized in CK19-positive biliary compartment at week 7 (W7) and later timepoints (Fig. 1D and Supplementary Fig. S2B). In concordance with these findings the number of EGFP+HNF4α+ hepatocytes decreased drastically during the postnatal liver maturation and by week 7 the majority of hepatocytes had lost their nuclear label (Fig. 1D,E). During the following weeks the pool of EGFP+ hepatocytes continued to decrease and by week 9–12 only extremely rare EGFP+HNF4α+ positive cells could be found after rigorous analysis of sections (Fig. 2B, arrow). At W15 no EGFP+HNF4α+ hepatocytes could be identified. The number of EGFP+CK19+ biliary cells decreased also during the first 7 weeks of observation albeit at much slower pace (from 86% on P5 to 71% at W7; Fig. 1E) and stabilized from W7 onwards demonstrating the establishment of the LLRC compartment in the biliary epithelium. Interestingly, the proportion of proliferative cholangiocytes was similar to that of the hepatocytes at all time points analyzed (Supplementary Fig. S1C,D), showing that the formation of the LLRC population was not caused by lower overall proliferation rate in the biliary compartment relative to the hepatocyte compartment. Furthermore, a comparative analysis of Ki67 expression in the biliary EGFP+ and EGFP− compartments demonstrated that EGFP+ cells did proliferate during liver maturation, however, their proliferative activity declined sharply at week 2 and remained lower than that of EGFP− cells during the next 12 weeks (Supplementary Fig. S3A–C).

To eliminate the possibility that the EGFP signal resulted from unspecific activation of H2B-EGFP expression, we analyzed the livers of the bigenic mice that did not receive dox. No EGFP− positive cells were detected in these livers, showing that the observed H2B-EGFP expression was exclusively dox-dependent (Supplementary Fig. S4).

To further characterize the cellular identity of LLRCs we stained liver sections with antibodies recognizing epithelial cell adhesion molecule (EpCAM), CD133, and CD166 that label biliary cells and have been used to isolate liver progenitor cells previously (ref. 3 and references therein). CK19 and HNF4α antibodies were used to identify cholangiocytes and hepatocytes, respectively (Fig. 2A,B). As expected, LRCs were positive for EpCAM (Fig. 2C; Supplementary Fig. S5A); in addition, LRCs expressed CD133 and increased levels of CD166 (Fig. 2D,E and Supplementary Fig. S5B,C) confirming their cholangiocyte identity. Furthermore, majority of the LLRCs were positive for A6 antibody, that has been used to label both biliary epithelium and oval cells^29^ (Fig. 2F and Supplementary Fig. S5D).

### Liver LRCs are activated in response to biliary tract injuries

**Figure 3:**
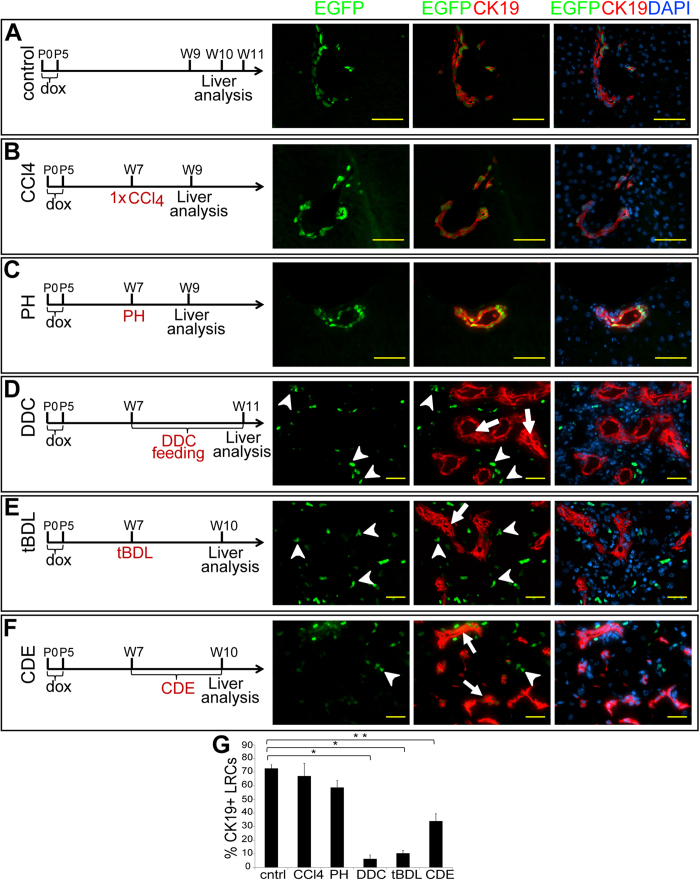
LLRCs are activated in response to biliary injury. CCl_4_ intoxication (**B**) or partial hepatectomy (PH) (**C**) did not alter the number of LRCs (green) in the CK19-expressing (red) bile ducts. DDC-feeding (**D**) and total bile duct ligation (tBDL) (**E**) induced ductular reaction (arrows), and the dilution or complete loss of EGFP signal in bile ducts. CDE diet (**F**) induced small ductular reactions. Simultaneously, small EGFP+ and CK19− cells (arrowheads) appeared around bile ducts (**D,E,F**). Uninjured livers were used as control (**A**). Nuclei (DAPI, blue). Scale bars: A–C 50 μm, D–F 25 μm. (**G**) The CK19+EGFP+ LLRC proportions were dramatically reduced in response to tBDL and DDC-diet (10% and 6% respectively, *p < 0,0001) and significantly reduced after CDE diet (35%, **p < 0,05) but not after PH or CCl_4_-treatment. n = 7 (control), n = 4 (CCl_4_), n = 4 (PH), n = 3 (DDC), n = 5 (tBDL), n = 2 (CDE). Data represent mean ± SEM.

Next we utilized six different liver damage models to study whether LLRCs are induced to proliferate in response to liver damage and whether these cells participate in liver repair. First, we induced acute liver damage in 7 week-old mice by a single i.p. injection of CCl_4_ that damages primarily hepatocytes, thus inducing regenerative response in this compartment^30^, and allowed the liver to recover for 14 days. CCl_4_ treatment did not significantly reduce the number of biliary EGFP^+^ cells when compared to untreated liver (Fig. 3A,B,G). Next we performed 2/3 partial hepatectomy (PH) to study the contribution of LLRCs to liver regeneration after massive hepatocyte loss. Mice were examined 14 days after surgery, which is a sufficient time period for liver regeneration^31^. Similarly to CCl_4_ treatment, the percentage of LLRCs in the bile ducts remained virtually unaltered (Fig. 3C,G) displaying only a slight tendency towards decrease. This finding is in good correlation with the notion that in addition to massive hepatocyte proliferation PH induces modest proliferative activity also within the biliary compartment^32^. These results show that LLRCs residing in biliary tracts are not induced to proliferate when recovery of hepatocytes is primarily required.

Obstruction of biliary flow by ligation of the common bile duct (total bile duct ligation–tBDL), feeding with 3,5-diethoxycarbonyl-1,4- dihydrocollidine (DDC) or choline-deficient, ethionine-supplemented (CDE) diet impose trauma primarily to biliary epithelial cells^33^,^34^,^35^. In order to study the LLRC activation in response to bile duct injury, mice were either fed with DDC-supplemented chow for 4 weeks, administered CDE-diet for 3 weeks or subjected to tBDL and the liver was examined 3 weeks after surgery. All injuries induced the biliary cells, including LRCs, to proliferate as judged by the appearance of numerous CK19-positive ductular reactions - the hallmarks of bile duct regeneration (Fig. 3D–F). Concomitantly, the number of LLRCs in bile ducts of treated mice was substantially decreased (Fig. 3G). The CDE-induced decrease was less dramatic, albeit significant.

**Figure 4:**
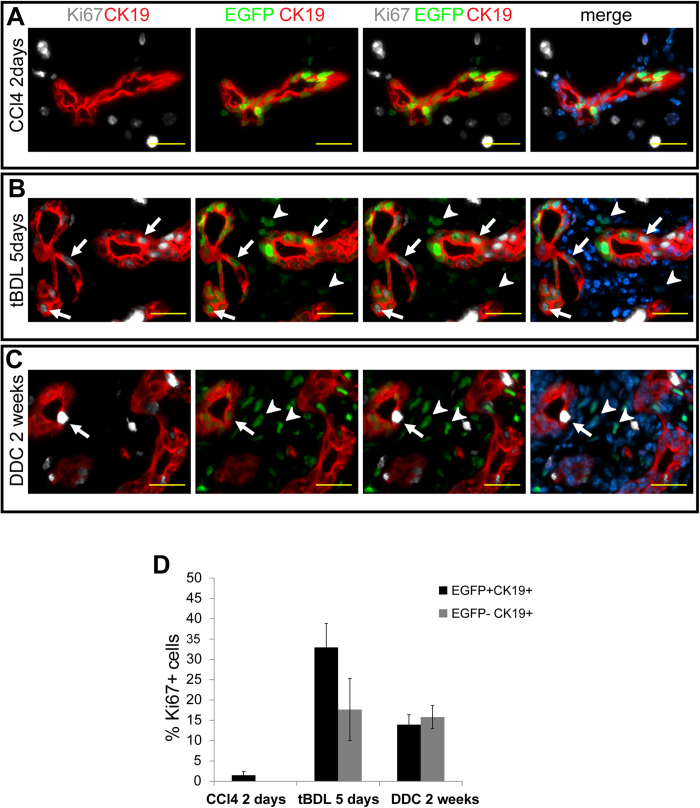
Proliferative status of biliary compartment in response to different types of liver injuries. (**A**) Proliferating cells (Ki67 +, grey) outside the CK19+ biliary compartment (red) 2 days after CCl_4_ injection. (**B**) Proliferating EGFP+CK19+Ki67+ cells (arrows) in biliary ducts 5 days after total bile duct ligation (tBDL). Arrowheads indicate infiltrating leukocytes. (**C**) Most cholangiocytes had lost their EGFP expression after 2 weeks of DDC feeding. Few EGFP+CK19+Ki67+ proliferating bile duct cells were detected (arrow). Arrowheads indicate infiltrating leukocytes. Nuclei (DAPI, blue). Scale bars: 25 μm. (**D**) Proliferation rate in biliary EGFP+ and EGFP− cells after different types of liver injuries. n = 2 per injury. Data represent mean ± SEM.

To study the proliferation dynamics within the biliary compartment we analyzed the proportion of proliferative LLRC (Ki67+EGFP+) and non-LRC (Ki67+EGFP−) cells in livers subjected to either hepatocyte (CCl_4_) or biliary (tBDL, DDC) injuries at time points with an ongoing active proliferative response. No significant increase in proliferation rate was detected in biliary ducts subjected to CCl4-mediated hepatocyte damage (Fig. 4A,D). In contrast, the LLRC and non-LRC biliary cells were induced to proliferate in response to biliary injuries (Fig. 4B,C). Interestingly, in case of tBDL the percentage of proliferating LLRCs was higher than that of non-LRC biliary cells while DDC induced proliferation equally in both cell populations (Fig. 4D). These data demonstrate that LLRCs were induced to proliferate only when bile duct injury was present.

Interestingly, as the number of LRCs in bile ducts decreased we could detect CK19-negative cells with strong EGFP expression in the vicinity of ductular reactions (Fig. 3D–F, arrowheads). The fact that H2B-EGFP transgene is promiscuously expressed in hematopoietic cells in the bone marrow without dox treatment^36^ led us to hypothesize that these EGFP+ cells might represent infiltrating cells of hematopoietic origin. Immunostaining of liver sections clearly demonstrated that the CK19-EGFP+ cells in injured livers express CD45 and, thus, originate from the hematopoietic system (Supplementary Fig. S6A). Furthermore, examination of the spleen of bigenic mice that had never received dox revealed EGFP+ cells in the marginal zone (Supplementary Fig. S6B) corroborating our data about the presence of EGFP+ hematopoietic cells in livers subjected to biliary damage.

### CK19-expressing biliary cells contribute to the regeneration of bile ducts but not parenchymal hepatocytes

**Figure 5:**
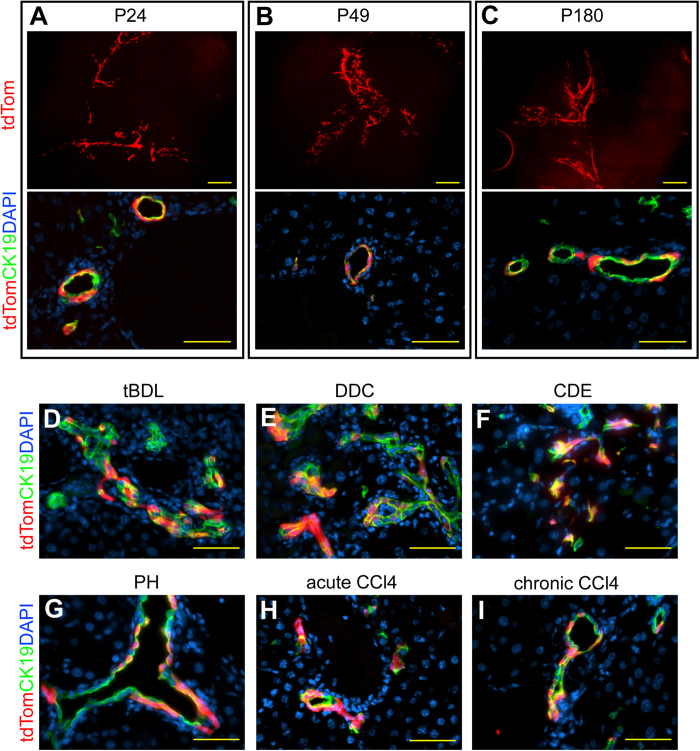
CK19+ cells participate only in biliary regeneration. (**A**–**C**) tdTomato (tdTom, red) expression in K19^CreERT2^/tdTomato mice 3 days (**A**), 1 month (**B**) and 5 months (**C**) after TMX injection. Upper image: tdTom (red) in biliary tree (1 mm-thick liver sections). Lower image: tdTom (red) co-expression with CK19 (green). Nuclei (DAPI, blue). Scale bars: upper image 200* *μm, lower image 50 μm. n = 3 per time point. (**D–I**) tdTom (red) expression after total bile duct ligation (tBDL) (**D**), DDC feeding (**E**), CDE diet (**F**), partial hepatectomy (PH) (**G**), acute CCl_4_ intoxication (**H**) and chronic CCl_4_ injury (**I**). CK19 (green). Nuclei (DAPI, blue). Scale bars: 50 μm. n = 3 (tBDL, DDC, PH, CCl_4_), n = 2 (CDE).

The collected data strongly suggested that LLRC located in the bile ducts were restricted in their differentiation potential to biliary lineage. To study the contribution of biliary cells to liver maintenance and regeneration we bred mice expressing a fusion protein of Cre recombinase and truncated estrogen receptor 2 (CreERT2) under the control of CK19 promoter (K19^CreERT2^) with a mouse line harboring a tdTomato (tdTom) expression cassette in Rosa26 locus preceded by a loxP-flanked STOP signal. Upon tamoxifen (TMX) injection the STOP signal is removed and tdTom is expressed in the particular cell and its progeny. We injected K19^CreERT2^/R26 -tdTom bigenic pups with TMX at P21 and followed the normal liver maintenance for 6 months (up to P180). 3 days post injection the tdTom-expressing cells could be found in CK19+ bile ducts whereas no tdTom-expressing hepatocytes could be identified (Fig. 5A). At P49 and P180 the tdTom expression was still restricted to the biliary system and tdTom labeled hepatocytes could not be detected even at P180 (Fig. 5B,C) indicating that in homeostasis the cholangiocytes do not participate in hepatocyte maintenance. To study the potential contribution of biliary epithelium to liver regeneration, we subjected TMX-injected mice to tBDL, DDC diet and CDE diet-induced biliary injury or CCl_4_ and PH mediated hepatocyte amelioration. As expected, tBDL, DDC diet and CDE diet induced tdTom-labeled ductular reactions but no hepatocytes derived from biliary compartment could be identified (Fig. 5D–F). Likewise, PH and acute CCl_4_-injury did not cause accumulation of tdTom-labeled hepatocytes (Fig. 5G,H). To induce long-term hepatocyte damage we administered CCl_4_ repeatedly during 4 weeks (Fig. 5I). Nevertheless, the tdTom-labeled cells of biliary origin could be found only within the biliary compartment and no contribution of CK19+ cells to hepatocyte compartment could be seen, indicating that biliary cells, including LLRCs, are restricted to biliary lineage in normal liver and in diverse liver damage models *in vivo*.

### LLRCs exert enhanced self-renewal properties but unipotent differentiation potential *in vitro*

**Figure 6:**
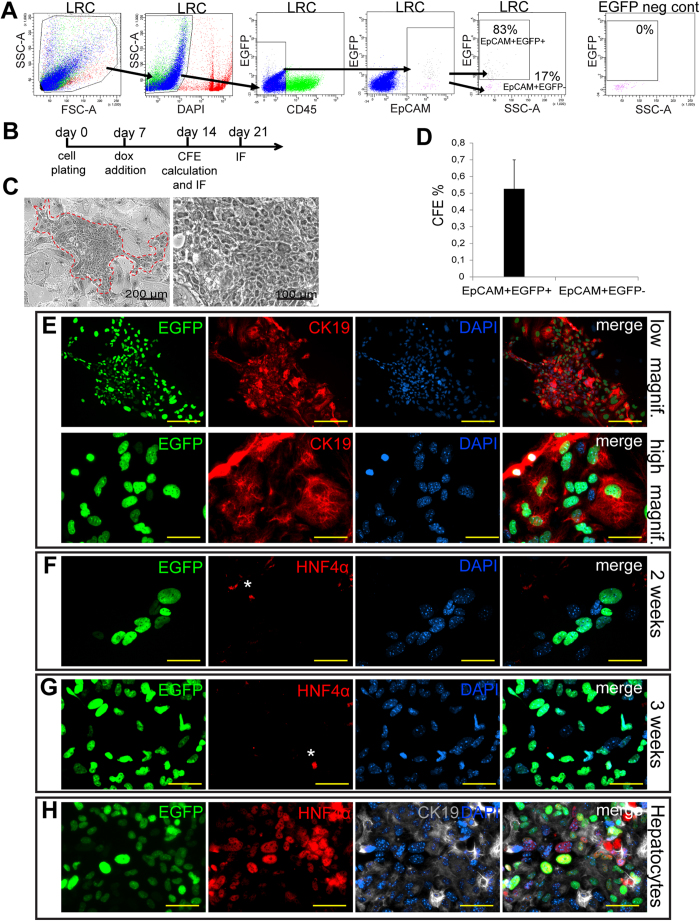
LLRCs possess enhanced unipotent progenitor properties *in vitro.* (**A**) Sorting strategy for EpCAM+EGFP+ and EpCAM+EGFP− cell isolation. Numbers indicate mean percentage of 4 independent experiments. CBA mouse was used as control. (**B**) Experimental setup for *in vitro* cultivation. (**C**) EpCAM+EGFP+ cell colony on feeder. (**D**) Colony forming efficiency (CFE) of EpCAM+EGFP+ and EpCAM+EGFP− cells. Data represent mean ± SEM of 4 independent experiments. *p < 0.05. (**E**–**G**) LLRC colonies express EGFP (green) and CK19 (red) (**E**) but not HNF4α (red) after 2-week (**F**) or 3-week (**G**) cultivation. Scale bars: E(upper panel) 200 μm, E (lower panel)-G 50 μm. *Non-specific staining. Experiment was performed 4 times. (**H**) Hepatocyte colonies show EGFP (green), HNF4α (red) and CK19 (grey) expression. Nuclei (DAPI, blue). Scale bars: 50 μm. Experiment was performed twice with similar results.

Self-renewal and multipotency are two key properties of somatic stem cells. Since isolated liver progenitor cells have been shown to differentiate into cholangiocytes and hepatocytes in culture^37^,^38^ we set out to evaluate the self-renewal capability and multipotency of normal mouse LLRCs *ex vivo*. Two cell populations -LLRCs (EGFP+EpCAM+CD45−) and non-LRC biliary cells (EGFP−EpCAM+CD45−) -were isolated from pulse-chased R26rtTA-H2B-EGFP mice using FACS sorting (Fig. 6A). The viability of sorted EGFP+ and EGFP− cells was comparable (Supplementary Fig. S7). Subsequently, equal numbers of live cells were seeded to irradiated feeder and 7 days after plating dox was added to the growth medium to re-induce H2B-EGFP expression and visualize the cells of bigenic mouse origin on feeder background (Fig. 6B and Supplementary Fig. S8). After two weeks of cultivation the EGFP+EpCAM+CD45− LLRCs formed colonies (Fig. 6C) that were readily detectable by EGFP expression (Fig. 6E–F), however, the EGFP-EpCAM+CD45− cells did not produce any colonies (Fig. 6D) suggesting that LLRCs harbored enhanced *in vitro* proliferation capacity when compared to non-LRC biliary cells. The colonies formed by LLRCs were positive for cholangiocyte markers CK19 (Fig. 6E) and Sox9 (Supplementary Fig. S8B) but were negative for hepatocyte markers HNF4α (Fig. 6F) and albumin (ALB) (Supplementary Fig. S8C). Even after prolonged, 3 week culturing no HNF4α+ cells appeared in the colonies formed by LLRCs (Fig. 6G). As expected, hepatocytes isolated from the same mice readily formed colonies that contained HNF4α-, ALB- and CK19-expressing cells (Fig. 6H and Supplementary Fig. S8D). These data show that despite the enhanced self-renewal properties, the LLRCs are restricted in their differentiation potential to biliary lineage under the culturing conditions used and support our notion that LLRCs exhibit unipotent biliary progenitor properties.

### LLRCs are defined by a unique gene expression profile

To uncover the molecular features that distinguish LLRCs from non-LRC biliary cells we compared the transcriptional profiles of sorted EGFP+EpCAM+CD45− LLRCs with EGFP−EpCAM+CD45− cholangiocytes using RNASeq. Stringent data analysis revealed 95 differently regulated genes, of these 20 were upregulated in LLRCs while 75 were downregulated (Supplementary Table S3). Nearly half of the genes upregulated in LLRCs were related to tumorigenesis (Spdya, Styk1, Fgr, Pbk, Palb2, Cxcl10, Tmc7, Mas1). Surprisingly, a number of upregulated genes function primarily in the central nervous system (Rab39b, ZFP711, Pigw, Slc1a4, Oprk1). The only known non protein-coding transcript in the list–Panct2–is involved in the regulation of pluripotency^39^. Interestingly, 40 of 75 downregulated genes were associated with plasma membrane and several of these were important in forming cell-cell contacts (eg Cldn5, Gja4, Esam, Pcdh12, Cdh5, Jam2, Cdh13, Pecam1, Eng). Notably, the genes related to vessel formation (eg Wnt2, Bmp4, Rspo3, Flt1, Tek) were downregulated in LLRCs suggesting their relative inactivity in respect of biliary system maintenance and a specific interaction with the surrounding environment when compared to non-LRC biliary cells.

## Discussion

In this work we took advantage of a genetic cell labeling approach to mark label-retaining cells established during normal development in mouse liver and characterized their potential as liver stem/progenitor cells. Our initial finding that the LLRCs were confined to cholangiocyte compartment was well in accord with a previous study that demonstrated the presence of LLRCs in portal regions of mouse liver^26^.

Further functional evaluation of LLRCs revealed that these cells were induced to proliferate when bile duct injury was present and lineage-tracing experiments excluded any contribution from cholangiocytes towards hepatocyte compartment in 6 different well-characterized liver damage models. These observations corroborate the recently published studies, which showed that new hepatocytes arising during homeostatic liver maintenance or after liver injury were derived from pre-existing hepatocytes and did not originate from cholangiocytes or mesenchymal cells^4^,^5^. The unipotency of LLRCs was further exemplified by the finding that despite of their self-renewal capability in culture the LLRCs failed to produce bilineal progeny.

Our experiments do not completely rule out the possibility that LLRCs are capable of differentiating into hepatocytes *in vitro* given that particular culture conditions, specifically designed for hepatocyte differentiation, are provided but clearly point to the fact that among the LLRCs there are no cells that would readily take the hepatocyte fate under the culture conditions that support the growth of normal differentiated hepatocytes. Interestingly, the cultivated hepatocytes formed colonies that contained cells bearing both cholangiocyte and hepatocyte markers suggesting that if present, the potential bipotent liver progenitors reside rather within the hepatocyte compartment. This possibility is further supported by notions that during liver regeneration the cells originating from the hepatocyte compartment acquired a bipotent phenotype, expressed cholangiocyte/liver progenitor markers and contributed to biliary epithelium, albeit at a low frequency^6^,^7^,^9^. The fact that bipotent hepatocyte-derived cells express cholangiocyte markers during liver regeneration might have partially contributed to the long-standing belief that potential bipotent liver progenitors reside or originate from the biliary compartment^40^,^41^,^42^,^43^,^44^.

While our work focused on LLRCs that were established during normal liver postnatal development, the role and properties of liver LRCs established during liver injury might well differ. In two earlier reports such LRCs localized in bile ducts but also in pericentral areas of liver lobule^25^,^26^. To date it is not clear whether the pericentral LRCs differ in their regenerative capacity from bulk hepatocytes. However, the recent discovery that Axin2-expressing diploid pericentral hepatocytes maintain the hepatocyte compartment^45^ warrants for further studies on the role of pericentral LLRCs in liver regeneration.

The close cellular identity of biliary LLRCs and non-LRC cholangiocytes is reflected in a moderate number of differently expressed genes. Surprisingly, a subset of genes upregulated in LLRCs (Rab39b, Zfp711, Pigw, Slc1a4) play a role in different forms of mental retardation^46^,^47^,^48^,^49^. Since Oprk1, an opioid receptor subunit, which is overexpressed in liver metastasis of colorectal cancer was also increased in LLRCs, one could speculate that a subset of signaling pathways active in neurons also define the specific properties of LLRCs^50^. In line with this, Gabrb1, a subunit of type A GABA receptor was the top downregulated gene in LLRCs and GABA signaling has been implicated in regulation of liver regeneration and tumorigenesis previously (reviewed in ref. 51).

Taken together, our experiments demonstrated that the regenerative capacity of LLRCs that are established during normal liver postnatal development is restricted to one tissue compartment–the bile ducts unlike that of the multipotent LRCs found in a number of other tissues.

## Materials and Methods

### Mice and treatments

H2B-EGFP mice, Rosa26-rtTa mice and Rosa26-tdTomato mice were obtained from the Jackson Laboratory. K19^CreERT2^ mice were kind gift from Guoqiang Gu and Cedric Blanpain. Both male and female mice with mixed background (C57BL/6 and CBA) between the ages of 5 days and 6 months were used in the experiments. Nursing mothers of bigenic R26-rtTa-H2B-EGFP pups received doxycycline in the drinking water (2 g/l, AppliChem), supplemented with 5% sucrose. At P21 the K19^CreERT2^/R26-tdTomato mice were injected intraperitoneally with 2 mg of tamoxifen in rapeseed oil. All procedures involving animals were conducted according to the guidelines approved by the Commission of Laboratory Animal Licenses at the Estonian Ministry of Agriculture (license no 25 and no 88).

### CCl_4_ injury

For acute parenchymal liver damage mice received a single intraperitoneal injection of CCl_4_ (1 μl/g body weight, Acros Organics) diluted in rapeseed oil. Liver samples were collected 2 days or 2 weeks later. To induce chronic CCl_4_ liver damage, mice received CCl_4_ injections (0.5 μl/g body weight) 3 times per week for 4 weeks (12 injections in total). Mice were sacrificed 2 days after last injection.

### DDC diet

For chronic hepatobiliary injury mice were fed 0.1% DDC-supplemented diet (3,5-diethoxycarbonyl-1,4-dihydrocollidine, Ssniff Spezialdiäten GmbH) for 2 or 4 weeks.

### CDE diet

Mice were fed choline-deficient diet (Altromin) and 0.15% DL-ethionine in the drinking water (Sigma-Aldrich) for 3 weeks.

### Partial hepatectomy

2/3 partial hepatectomy (PH) was performed as previously described in ref. 52. Two weeks after surgery the regenerated livers were harvested.

### Total bile duct ligation (tBDL)

The common bile duct was double ligated with suture (5/0, B.Braun) and cut between ligatures. The experiment was ended 5 days, 2 weeks or 3 weeks later and livers were harvested.

### Sample freezing

Liver samples of R26-rtTA-H2B-EGFP mice were embedded in O.C.T compound (Tissue-Tek), frozen in cold isopentane and stored at −80 °C. Livers of K19^CreERT2^/tdTomato mice were first perfused with PBS and then with 4% paraformaldehyde via portal vein. Fixed liver samples were placed in 30% sucrose solution for 2 hours at 4 °C. Finally, the liver samples were embedded and frozen as described above. 5–7 μm-thick frozen sections were cut for immunofluorescence.

### Immunofluorescence

Tissue sections and cells were fixed with 4% paraformaldehyde and permeabilized with 0.3% Triton X-100. After blocking with 4% normal donkey serum, the slides were incubated with primary antibodies overnight at 4 °C, followed by incubation with secondary antibodies. Nuclei were counterstained with DAPI (0.1 μg/ml, Sigma-Aldrich). Antigen retreival of pre-fixed frozen sections was performed by incubating slides in 10 mM Na-citrate buffer (pH 8.7) for 30 minutes at 80 °C prior permeabilization and subsequent antibody staining. Antibodies are listed in Supplementary Tables S1 and S2 in Supplementary Materials and Methods.

### Isolation of parenchymal and non-parenchymal cells (NPCs) from mouse liver

Cells were isolated by two-step perfusion protocol modified from^53^. Briefly, under anesthesia the mouse liver was perfused via portal vein with EGTA solution at 4 ml/min for 5 minutes, followed by perfusion with Collagenase type II solution (0.35 mg/ml, Gibco) for 15 minutes. Next, the liver was excised and cells were dissociated in Williams medium E supplemented with 5% FBS by vigorous shaking and careful pipetting. The cell suspension was filtered through 70-μm strainer. The remaining undissociated liver tissue was further incubated in Pronase solution (0.5 mg/ml) for 5 minutes at 37 °C and the aquired cell suspension was combined with cells from the previous step. Hepatocytes were removed by repeated centrifugation at 50 × g for 1 minute. Supernatant was further centrifuged at 300 × g for 10 minutes and the pellet containing NPCs was incubated in red blood cell lysis buffer (BD Pharm Lyse^TM^ Lysing Buffer, Becton Dickinson) for 10 minutes on ice. CD45 antibody-coupled MicroBeads were used for leukocyte depletion according to the manufacturer’s protocol (Miltenyi Biotech). Obtained cell suspension was further used for cell sorting. Hepatocytes were washed and collected for plating.

### Cell sorting, plating and cultivation

NPC suspension was blocked with 1% BSA and then incubated with EpCAM-APC (1:100) and CD45-PE (1:1000) antibodies for 1 hour on ice. Dead cells were excluded by DAPI staining (0.5 μg/ml). NPCs obtained from CBA mice were used as controls for EGFP. Cells were sorted into Williams E medium supplemented with 20% FBS and 10 μM ROCK inhibitor (Y-27632, Tocris). Cell sorting was performed with FACS Aria Cell Sorter (Becton Dickinson). Sorted cells were centrifuged at 300 × g for 5 minutes and plated in growth medium supplemented with 10 μM ROCK inhibitor. Cells were seeded on collagen I coated 8-well chamber slides (Corning) covered with irradiated MEF feeder (mouse embryonic fibroblasts). In 48 h new medium without ROCK inhibitor was added. Doxycycline (200 ng/ml) was added to the medium 1 week later. Cells were cultured for three weeks and the medium was changed every three days. Hepatocytes were plated on collagen I coated 8-well chamber slides and cultivated as described above. Further details on used solutions and medium components are provided in Supplementary Materials and Methods.

### RNA analysis

Cells were prepared for FACS sorting as described above and sorted into RNA Extraction buffer. RNA was extracted with PicoPure^TM^ RNA Isolation Kit (Arcturus) according to manufacturer´s protocol. The RNAseq analysis and read mapping was performed as a service at EMBL. HT-seq python package (version, 0.5.3p9, with flags–mode = union–stranded = no–type = exon) was used to count the number of reads aligned to the reference genes (genome build GRCm38.p4, Ensembl). Data were uploaded to Gene Expression Omnibus (GEO, accession no. GSE90160). Differential gene expression analysis was done with R using package EdgeR (v3.2.4) and a gene was considered differentially expressed if the adjusted p-value was <0.05. Gene ontology analysis was performed using PANTHER gene analysis tool (pantherdb.org). RNA analysis data are available in Supplementary Table S3.

### Colony forming efficiency (CFE)

The colonies were counted two weeks after plating based on EGFP expression. Colonies containing 10 cells or more were counted. CFE = colonies counted/cells plated × 100%. CFE was measured in four independent experiments.

### Statistics

Data are represented as mean ± SEM. Statistical significance was calculated using two-tailed unpaired Student’s t test. p < 0.05 was considered significant.

## Additional Information

**How to cite this article**: Viil, J. *et al*. A label-retaining but unipotent cell population resides in biliary compartment of mammalian liver. *Sci. Rep.* **7**, 40322; doi: 10.1038/srep40322 (2017).

**Publisher's note:** Springer Nature remains neutral with regard to jurisdictional claims in published maps and institutional affiliations.

## Supplementary Material

Supplementary Information
