# Supplementary material for: A label-retaining but unipotent cell population resides in biliary compartment of mammalian liver
Source: Sci Rep. 2017 Jan 13;7:40322. doi: 10.1038/srep40322 (PMC5234023; doi:10.1038/srep40322)
Supplement: Supplementary Information [file srep40322-s1.pdf]

Supplementary material

A label-retaining but unipotent cell population resides in biliary compartment of mammalian liver.

Authors: Janeli Viil[1], Mariliis Klaas[1], Kadri Valter[1], Denis Belitškin[1], Sten Ilmjärv[2] and Viljar Jaks[1, 3 *]

Affiliations: [1] Institute of Molecular and Cell Biology, University of Tartu, Tartu, Estonia. [2] Department of Pathology and Immunology, Medical School, University of Geneva, Geneva, Switzerland. [3] Karolinska Institutet, Stockholm, Sweden.

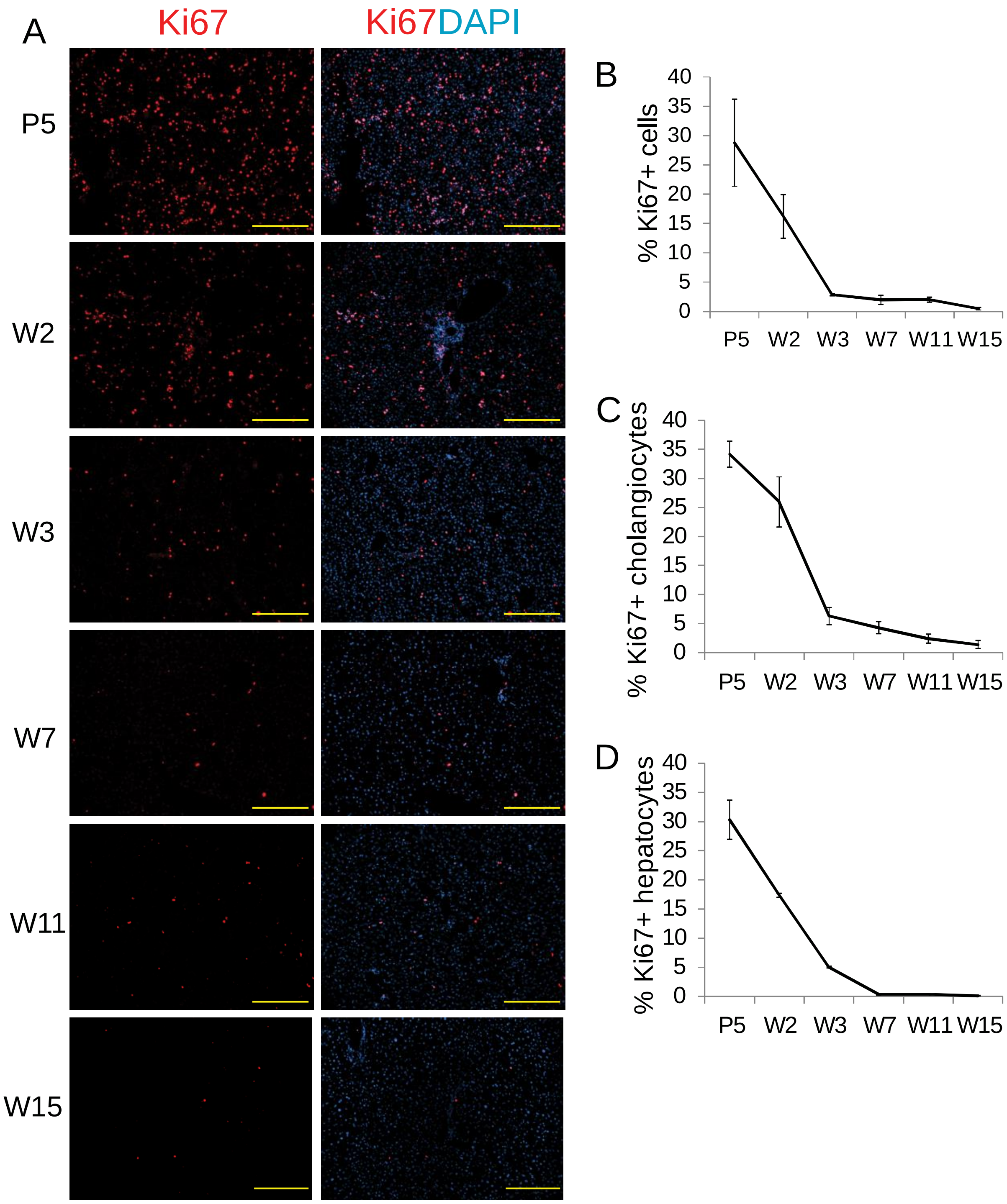

Supplementary Figure S1. Dynamics in liver proliferation rate during the postnatal liver maturation.

(A) Proliferating cells (Ki67+, red) in mouse liver at indicated time points. Nuclei are stained with DAPI (blue). Scale bars: 200 µm.

(B) Overall proliferation rate in the liver at different time points. n=2-3 per time point

(C-D) Proliferation rate of cholangiocytes (C) and hepatocytes (D) at different time points. n=2-3 per time point.

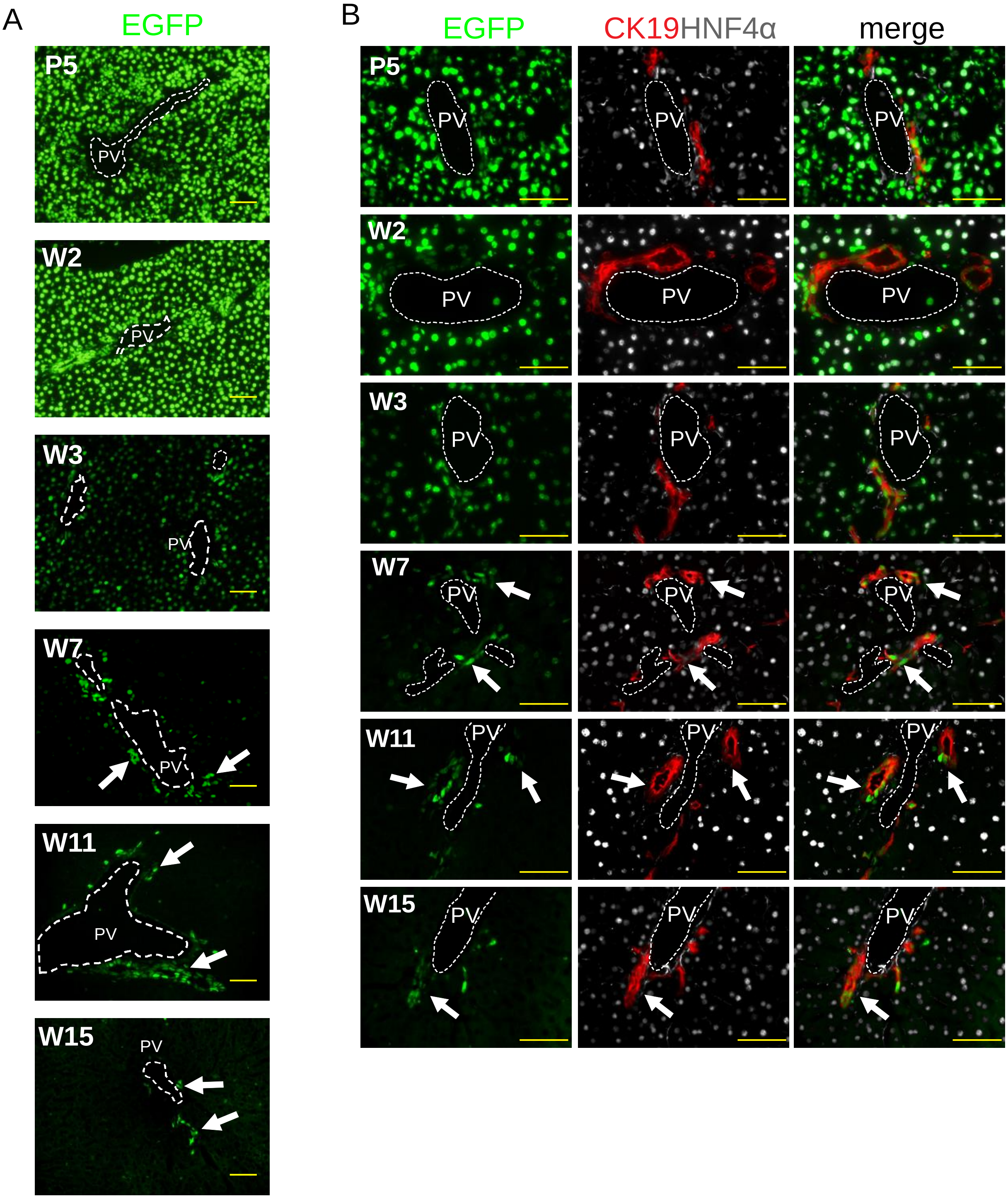

Supplementary Figure S2. Establishment of liver LRCs in portal areas during liver maturation.
(A) P5 and W2 livers show strong overall H2B-EGFP expression (green). From week 3, the EGFP signal is starting to dilute. Arrows indicate LRCs. Nuclei were stained with DAPI (blue). n=3 per time point
(B) Majority of EGFP+ LRCs (green) are concentrated around the portal vein and in the CK19-positive (red) bile ducts (arrows). Hepatocytes (HNF4α+, grey). Scale bars: 50 µm. PV-portal vein. n=3 per time point.

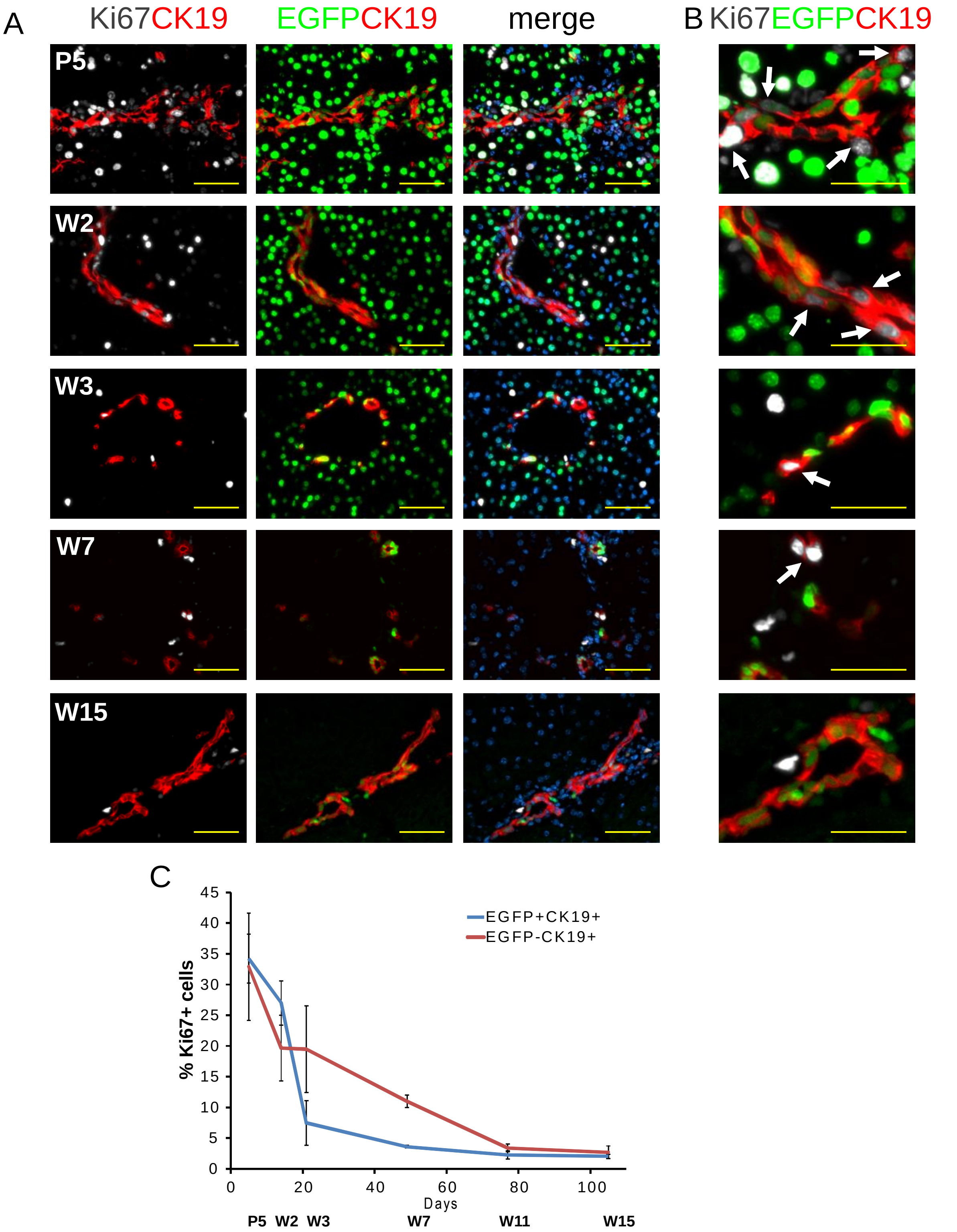

Supplementary Figure S3. Proliferation dynamics in biliary compartment during liver maturation.
(A-B) Proliferation of liver cells at different time points. Arrows indicate proliferating cholangiocytes (Ki67+, grey and CK19+, red). EGFP (green). Nuclei were stained with DAPI (blue). Scale bars: A 50 µm, B 25 µm.

(C) A comparative analysis of Ki67 expression in EGFP+ (blue) and EGFP- (red) cholangiocytes during homeostatic liver maintenance. Ki67 positive cells were counted in biliary EGFP+CK19+ and EGFP-CK19+ cells. n=2-3 per time point.

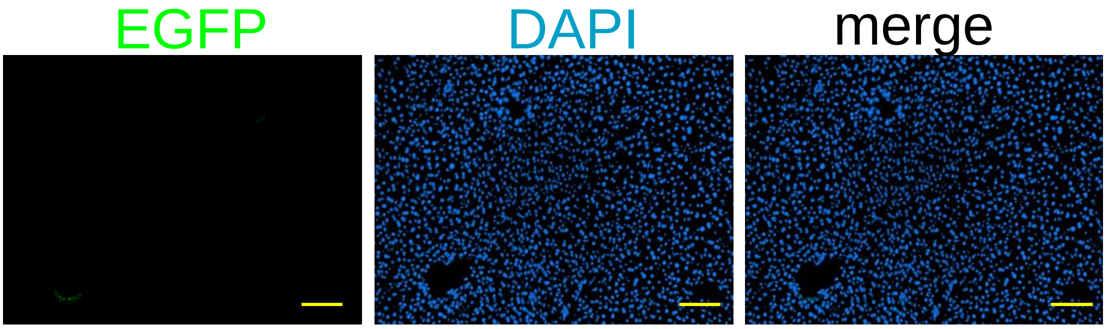

Supplementary Figure S4. H2B-EGFP expression without doxycycline induction in normal mouse liver.
No EGFP expression was detected in the livers of bigenic R26-rtTA-H2B-EGFP mice without doxycycline treatment. Nuclei were stained with DAPI (blue). Scale bars: 100 µm.

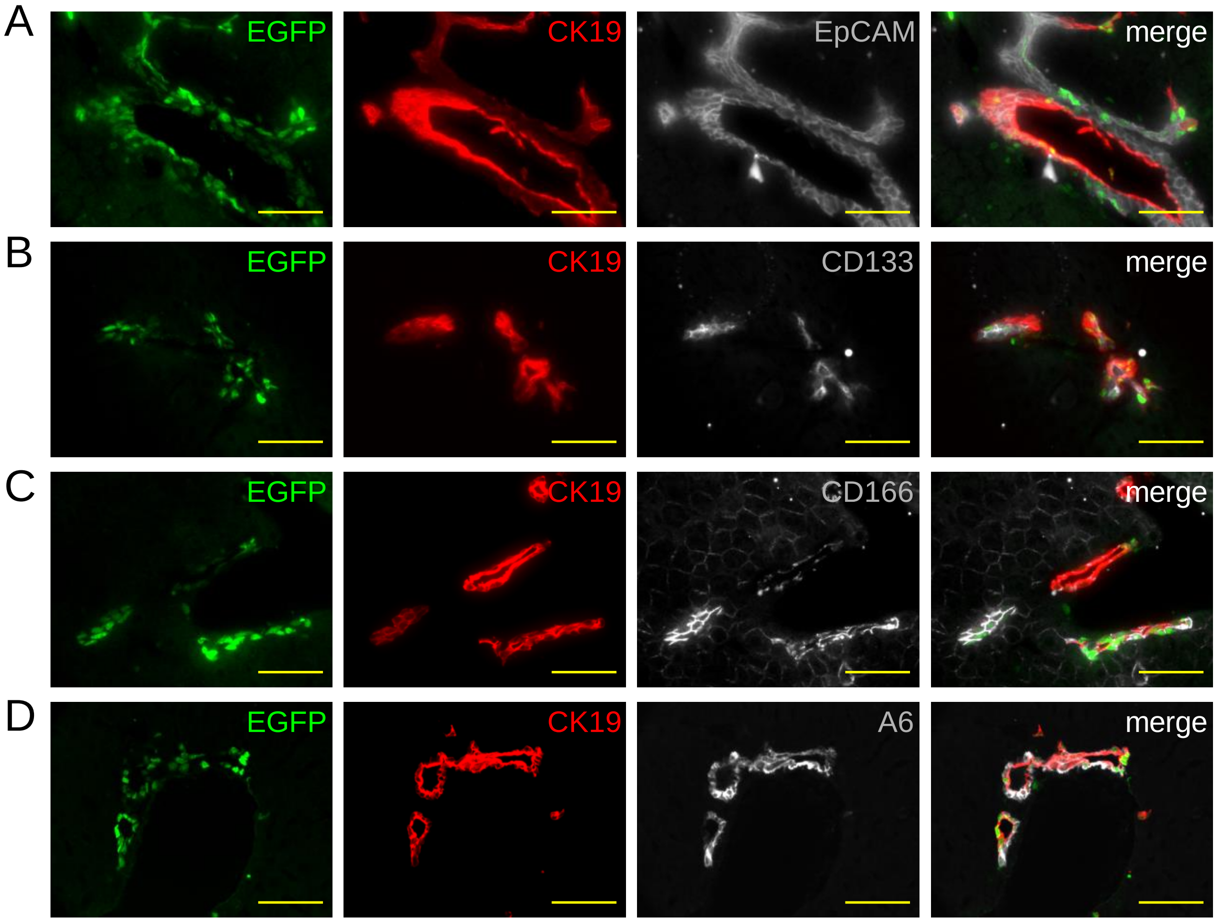

Supplementary Figure S5. Co-expression of biliary epithelial cell markers in LLRC compartment.
Cholangiocyte marker CK19 (red) is co-expressed with EpCAM (A, grey), CD133 (B, grey), CD166 (C, grey) and A6 (D, grey) in LRCs (green) in the liver. Scale bars: 50 µm.

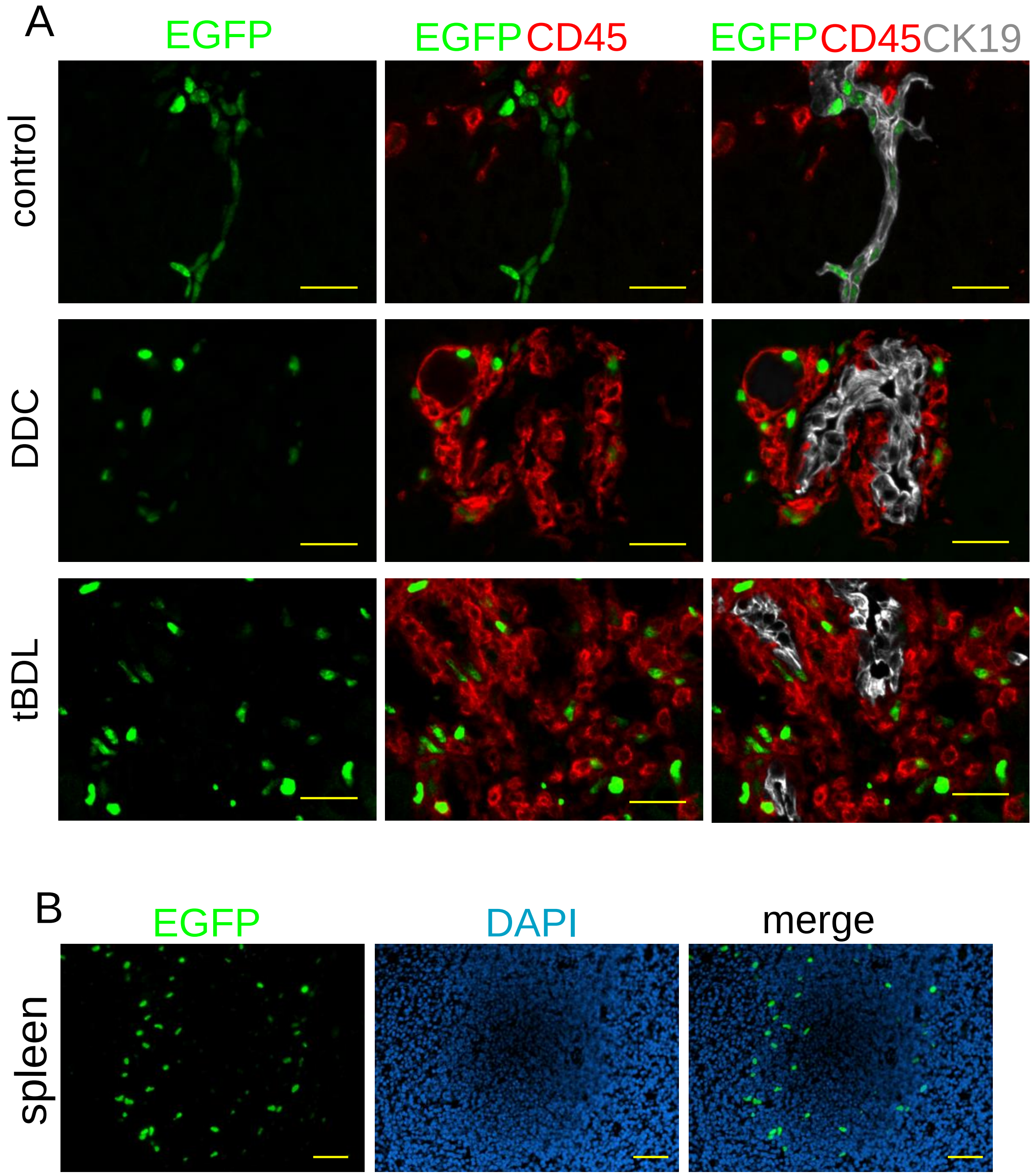

Supplementary Figure S6. Dox-independent H2B-EGFP expression in infiltrating hematopoietic cells.

(A) LLRCs (green) in normal control mouse were CD45- (red) and CK19+ (grey) while infiltrating EGFP+ (green) cells in injured livers were CD45+ and CK19- confirming their hematopoietic origin. Scale bars: 25 µm. tBDL- total bile duct ligation. n= 3 per injury.

(B) EGFP+ cells (green) in the spleen of bigenic R26-rtTA-H2B-EGFP mouse without doxycycline induction. Nuclei were stained with DAPI (blue). Scale bars: 100 µm.

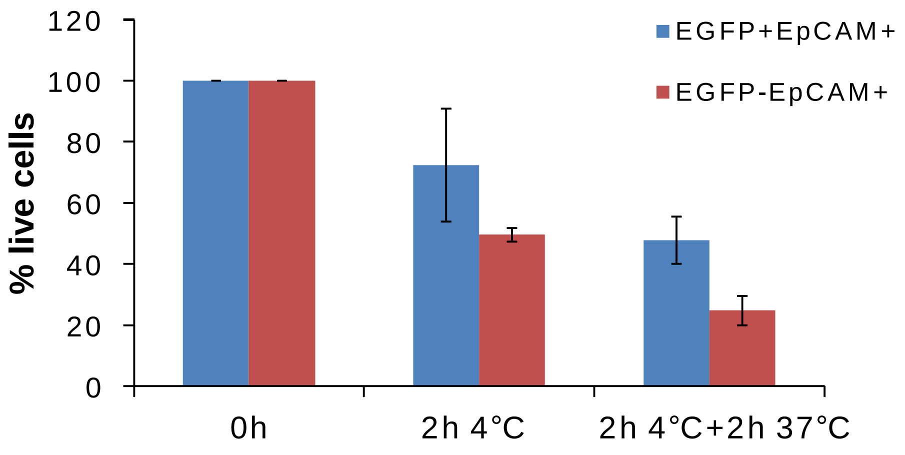

Supplementary Figure S7. Analysis of cell viability after FACS sorting.
Viability of sorted EGFP+EpCAM+ (blue) and EGFP-EpCAM+ (red) cells directly after sorting (0h), after 2-hour incubation on ice (2h 4°C), and after 2-hour incubation on ice and 2-hour incubation in suspension in cell incubator (2h 4°C+2h 37°C). n=2. Data represent mean ± SEM.

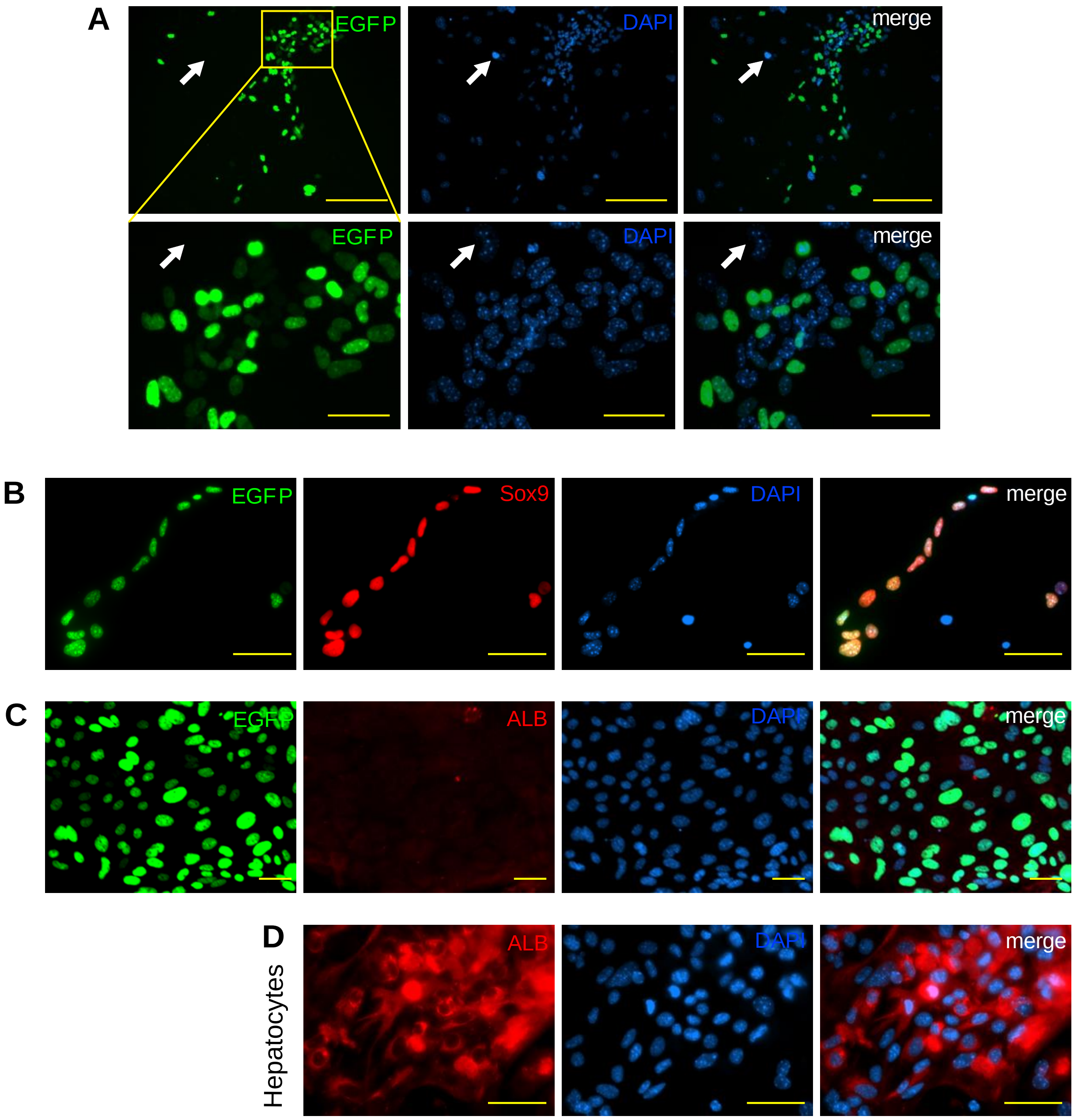

Supplementary Figure S8. H2B-EGFP expression labels cells derived from R26-rtTA-H2B-EGFP bigenic mice.
(A) Nuclear EGFP expression (green) in a cell colony derived from purified EpCAM+ EGFP+ cells. Feeder cells are EGFP- (arrow).
(B-C) LLRCs (green) express Sox9 (red) (B) but not ALB (C).
(D) ALB (red) expression in hepatocytes. Nuclei were stained with DAPI (blue). Scale bars: A (top row) 200 µm, A (bottom row)-D 50 µm.

Supplementary tables

Supplementary Table S1. List of primary antibodies

Antibody	Host, cat no	Dilution	Source, reference
Cytokeratin 19	Rabbit, 3863-1	1:20000	Epitomics
EpCAM (CD326)	Rat, 118201	1:200	BioLegend
HNF4α	Goat, sc-6556	1:200	Santa Cruz Biotechnology
CD133-PE	Rat,12-1331	1:100	eBioscience
CD166-PE	Rat, 12-1661	1:100	eBioscience
CD45-PE	Rat, 12-0451	1:1000	eBioscience
EpCAM-APC	Rat, ab95641	1:100	Abcam
A6	Rat	1:100	gift from Dr. Valentina Factor
Ki67	Rat,14-5698-82	1:200	eBioscience

Supplementary Table S2. List of secondary antibodies

Antibody	Cat no	Dilution	Source
Alexa Flour 594 Donkey anti-rabbit	A21207	1:1000	Invitrogen
Alexa Flour 594 Donkey anti-rat	A21209	1:1000	Invitrogen
Alexa Flour 594 Donkey anti-goat	A11058	1:1000	Invitrogen
Alexa Flour 647 Chicken anti-goat	A21469	1:1000	Invitrogen
Alexa Flour 647 Donkey anti-rabbit	A31573	1:1000	Invitrogen

Supplementary Table S3. List of genes differentially (FDR<0,05) expressed in liver LRCs when compared to non-LRC biliary cells.

geneID	logFC	FDR	Gene Name
ENSMUSG00000029212	10,21459939	0,048876854	Gabrb1
ENSMUSG00000050234	7,86332491	2,23E-05	Gja4
ENSMUSG00000034037	7,408444912	0,041488057	Fgd5
ENSMUSG00000027435	7,227174185	0,000593835	Cd93
ENSMUSG00000046916	6,582207378	2,03E-05	Myct1
ENSMUSG00000024440	6,576655861	0,01557578	Pcdh12
ENSMUSG00000026872	6,44943707	0,000129247	Zeb2
ENSMUSG00000048120	5,923759088	1,45E-06	Entpd1
ENSMUSG00000046280	5,914456762	0,000413124	She
ENSMUSG00000090958	5,871570824	0,005616017	Lrrc32
ENSMUSG00000053062	5,710581566	2,03E-05	Jam2
ENSMUSG00000035778	5,710228204	0,008177825	Ggta1
ENSMUSG00000031841	5,584604358	0,000102106	Cdh13
ENSMUSG00000022220	5,4206733	0,009068885	Adcy4
ENSMUSG00000022122	5,393082659	3,89E-05	Ednrb
ENSMUSG00000062515	5,389774697	0,000139266	Fabp4
ENSMUSG00000019880	5,312291626	0,002701976	Rspo3
ENSMUSG00000039167	5,310021571	0,000129247	Adgrl4
ENSMUSG00000038543	5,30583152	0,018411107	BC028528
ENSMUSG00000026712	5,276617172	1,05E-07	Mrc1
ENSMUSG00000039706	5,112524109	0,018255684	Ldb2
ENSMUSG00000024168	4,969675237	4,50E-05	Tmem204
ENSMUSG00000030123	4,898145162	0,01478131	Plxnd1

ENSMUSG00000037872	4,840395692	0,012224512	Ackr1
ENSMUSG00000001930	4,776744274	0,000129247	Vwf
ENSMUSG00000041445	4,703047804	0,000129247	Mmrn2
ENSMUSG00000054690	4,658098374	0,003613076	Emcn
ENSMUSG00000025666	4,648392129	0,004473572	Tmem47
ENSMUSG00000047867	4,636848351	0,001675993	Gimap6
ENSMUSG00000074206	4,442794394	0,0165118	Adh6b
ENSMUSG00000034845	4,356988356	0,000442891	Plvap
ENSMUSG00000020717	4,353659364	0,002701976	Pecam1
ENSMUSG00000025044	4,321804857	0,000593835	Msr1
ENSMUSG00000017309	4,321748411	0,004887139	Cd300lg
ENSMUSG00000056492	4,289079115	0,000650217	Adgrf5
ENSMUSG00000022579	4,279747068	0,001286036	Gpihbp1
ENSMUSG00000030653	4,235684418	0,001854733	Pde2a
ENSMUSG00000010797	4,231370229	0,012535227	Wnt2
ENSMUSG00000004655	4,101745847	5,01E-05	Aqp1
ENSMUSG00000043008	4,045462267	0,015892552	Klhl6
ENSMUSG00000029648	4,03815334	0,000593835	Flt1
ENSMUSG00000021835	4,018168404	0,006082133	Bmp4
ENSMUSG00000028108	4,010461458	0,005210479	Ecm1
ENSMUSG00000035493	3,883914818	0,045724867	Tgfbi
ENSMUSG00000062960	3,854838665	0,000290188	Kdr
ENSMUSG00000045930	3,850717209	0,000707785	Clec14a
ENSMUSG00000021186	3,833662717	0,000707785	Fbln5
ENSMUSG00000027074	3,831440714	0,000129247	Slc43a3
ENSMUSG00000041378	3,811967394	0,00761932	Cldn5
ENSMUSG00000020154	3,807845616	0,000364386	Ptprb
ENSMUSG00000024065	3,798038107	3,53E-05	Ehd3
ENSMUSG00000006386	3,731912752	0,000221085	Tek
ENSMUSG00000031196	3,652880499	0,006412106	F8
ENSMUSG00000026814	3,650984019	0,002123499	Eng
ENSMUSG00000001946	3,615669239	0,009068885	Esam
ENSMUSG00000020077	3,613138545	0,010092496	Srgn
ENSMUSG00000090272	3,602701035	0,020720157	Mndal
ENSMUSG00000045312	3,542244153	0,011964929	Lhfpl2
ENSMUSG00000059588	3,489928775	0,000139266	Calcrl
ENSMUSG00000009654	3,459509257	0,001419612	Oit3
ENSMUSG00000021759	3,452966453	0,000703105	Ppap2a
ENSMUSG00000025279	3,443778641	0,004430527	Dnase1l3
ENSMUSG00000028713	3,321293898	0,009403374	Cyp4b1
ENSMUSG00000048376	3,319322627	0,031978463	F2r
ENSMUSG00000031871	3,305709264	0,047703468	Cdh5
ENSMUSG00000074491	3,208158066	0,003649065	Clec4g
ENSMUSG00000025810	3,075189563	0,001675993	Nrp1
ENSMUSG00000028003	3,054285605	0,010014758	Lrat
ENSMUSG00000026656	2,868248113	0,040049184	Fcgr2b
ENSMUSG00000074743	2,687852892	0,023445821	Thbd
ENSMUSG00000020577	2,680307801	0,016104381	Tspan13
ENSMUSG00000038007	2,677492682	0,031978463	Acer2
ENSMUSG00000037852	2,613130206	0,010301892	Cpe
ENSMUSG00000016194	2,482191115	0,025881581	Hsd11b1
ENSMUSG00000026365	2,481857996	0,023539751	Cfh

ENSMUSG00000031202	-9,32960352	0,000791068	Rab39b
ENSMUSG00000052525	-8,906431564	3,89E-05	Spdya
ENSMUSG00000032899	-8,603938336	0,039929727	Styk1
ENSMUSG00000025529	-8,431258105	0,037632529	Zfp711
ENSMUSG00000070527	-8,002665399	0,001798539	Mkrn3
ENSMUSG00000045140	-7,946843058	0,002759248	Pigw
ENSMUSG00000028874	-7,680303592	0,003172496	Fgr
ENSMUSG00000020142	-7,639170061	0,004118334	Slc1a4
ENSMUSG00000104515	-7,609571196	0,030591854	Gm37163
ENSMUSG00000025905	-7,582980337	0,000102106	Oprk1
ENSMUSG00000022033	-7,461532127	0,006192005	Pbk
ENSMUSG00000044702	-7,182462569	0,023562987	Palb2
ENSMUSG00000097814	-7,039214606	0,000129247	Panct2
ENSMUSG00000034855	-7,007531774	0,023445821	Cxcl10
ENSMUSG00000086706	-6,922116022	0,035291992	Gm15848
ENSMUSG00000073274	-6,104747456	0,00306856	Gm14636
ENSMUSG00000042246	-5,73599692	0,002555431	Tmc7
ENSMUSG00000068037	-4,960396543	0,042529063	Mas1
ENSMUSG00000089940	-4,703500815	0,01225927	Gm4117
ENSMUSG00000089810	-4,520104161	0,010948614	Gm16536

LRCs: label-retaining cells; logFC-logarithmic fold change; FDR-false discovery rate. A gene was considered differentially expressed if the adjusted p-value (FDR) was <0.05. n=4

Supplementary Materials and Methods

Solutions and medium

EGTA solution: Krebs-Ringer buffer (119 mM NaCl, 4.7 mM KCl, 1.2 mM KH_2PO_4, 1.2 mM $MgSO_4$, 4.2 mM $NaHCO_3$, 2 mM $CaCl_2$, 10 mM glucose) supplemented with 3 mM EGTA.

Collagenase solution: Krebs- Ringer buffer supplemented with 1 mM $CaCl_2$ and Collagenase type II (0.35 mg/ml).

Pronase solution: Collagenase solution supplemented with pronase (0.5 mg/ml, Roche).

Growth medium: Williams medium E containing 5% FBS, 1µM dexamethasone, 1% penicillin/streptomycin, 4 µg/ml human recombinant insulin, 2 mM GlutaMax, 15 mM Hepes (pH 7.4), 1 mM sodium pyruvate, 1 x Insulin-Transferrin-Selenium-Ethanolamine, 50 ng/ml recombinant human EGF, 30 ng/ml recombinant human HGF (all Gibco).

Viability measurement

0,4%Trypan blue solution was added to the cell suspension (1:1). Live and dead cells were counted after 5 minutes of incubation. Viability=live/total x 100%

Image analysis

Images were obtained with Olympus BX61 and Olympus CellR fluorescence microscopes, and processed with Hokawo software v2.1 (Hamamatsu Photonics).
